# The associations between *CYP19A1* polymorphisms and serum 17ß‐estradiol levels in Chinese patients with Alzheimer’s disease

**DOI:** 10.1002/alz.086088

**Published:** 2025-01-03

**Authors:** Lu Hua Chen, Yan Hui Fan, You‐Qiang Song, Leung‐Wing Chu

**Affiliations:** ^1^ Faculty of Health and Social Sciences, The Hong Kong Polytechnic University, Hong Kong China; ^2^ Western Institute of Computing Technology, Chinese Academy of Sciences, Chongqing China; ^3^ Li Ka Shing Faculty of Medicine, The University of Hong Kong, Hong Kong China

## Abstract

**Background:**

Accumulating evidence has shown the neuroprotective effects of estrogen on cognition function, for example delaying the cognitive deterioration in patients with Alzheimer’s disease (AD). However, the clinical usage of estrogen in AD remains controversial. The cytochrome P450 aromatase encoded by *CYP19A1*, is a key enzyme catalyzing the C19 androgen conversion to C18 estrogen, which induces testosterone to estradiol and androstenedione to estrone. The aromatase thus has been suggested to be an important partner of estrogen by controlling estrogen biosynthesis. Although increasing evidence has demonstrated the associations between *CYP19A1* polymorphisms and AD susceptibility, previous studies are mainly carried out in Caucasian populations, little is known about the role of this gene and its impact on serum estrogen levels in Chinese population.

**Method:**

We recruited 689 subjects including both AD patients and non‐demented controls in Hong Kong. Subjects’ serum 17ß‐estradiol were measured based on a chemiluminescent immunoassay principle. Genomic DNA was isolated from the whole blood and subsequent genotyping for 27 SNPs was analyzed on high‐throughput Sequenom® genotyping platform according to the manufacturer’s protocols. Statistical analysis was done using SPSS and PLINK.

**Result:**

After quality control, 3 SNPs were filtered and 24 SNPs spanning 122kb of this gene were included in data analysis. There was no significant difference for serum17ß‐estradiol levels between AD patients and non‐demented controls. The *APOE* ε4 allele was consistently associated with an increased AD risk (p<0.001). While the distribution of *CYP19A1* polymorphisms was very similar between AD patients and non‐demented controls and no significant differences were observed for each individual polymorphism in the sample. However, using multiple linear regression analyses, we found significant associations between 4 (rs2008691, rs1902586, rs7181886, and rs17647719) *CYP19A1* polymorphisms with serum17ß‐estradiol levels (p = 0.004, 0.039, 0.046 and 0.047, separately) after permutation by 10,000 times in Chinese population.

**Conclusion:**

In conclusion, our findings suggest a novel link between *CYP19A1* polymorphisms and variation of serum 17ß‐estradiol levels in Chinese elderly. This novel evidence may reflect inter‐individual differences of estrogen exposure according to different *CYP19A1* genotypes during aging and dementia process. Our findings may highlight a new perspective to understand the underlying mechanisms of AD.

